# Assessment of the Kinetic Trajectory of the Median Nerve in the Wrist by High-Frequency Ultrasound

**DOI:** 10.3390/s140507738

**Published:** 2014-04-28

**Authors:** Yi-Hsun Lin, Mei-Yu Hsieh, Fong-Chin Su, Shyh-Hau Wang

**Affiliations:** 1 Department of Computer Science and Information Engineering & Institute of Medical Informatics, National Cheng Kung University, No. 1, University Road, Tainan City 70101, Taiwan; E-Mails: hsun224@gmail.com (Y.-H.L.); shyhhau@gmail.com (M.-Y.H.); 2 Department of Biomedical Engineering, National Cheng Kung University, No. 1, University Road, Tainan City 70101, Taiwan; E-Mail: fcsu@mail.ncku.edu.tw; 3 Medical Device Innovation Center, National Cheng Kung University, No. 1, University Road, Tainan City 70101, Taiwan

**Keywords:** high-frequency ultrasound, cross correlation, motion tracking, median nerve, carpal tunnel syndrome

## Abstract

Carpal tunnel syndrome (CTS) is typically diagnosed by physical examination or nerve conduction measurements. With these diagnostics however it is difficult to obtain anatomical information in the carpal tunnel. To further improve the diagnosis of CTS, an attempt using 30 MHz high-frequency ultrasound to noninvasively detect the local anatomical structures and the kinetic trajectory of the median nerve (MN) in the wrist was explored. Measurements were performed on the right wrist of 14 asymptomatic volunteers. The kinetic trajectory of the MN corresponding to flexion (from 0° to 90°) and extension (from 90° to 0°) movements of the fingers were detected by a cross correlation-based motion tracking technique. The average displacements of the MN according to finger movements were measured to be 3.74 and 2.04 mm for male and female subjects, respectively. Moreover, the kinetic trajectory of the MN in both the ulnar-palmar and total directions generally follows a sigmoidal curve tendency. This study has verified that the use of high-frequency ultrasound imaging and a motion tracking technique to sensitively detect the displacement and kinetic trajectory of the MN for the assessment of CTS patients is feasible.

## 1. Introduction

Carpal tunnel syndrome (CTS), one of the most common neuropathic diseases, is frequently caused by a chronic compression of the median nerve (MN) beneath the transverse carpal ligament of the wrist [1]. CTS can lead to several clinical symptoms, such as burning pain, numbness, and paresthesia in the distribution of the MN that involves the thumb, index finger, and long fingers [2]. CTS is usually assessed by physical examinations or electrophysiological measurements. Tinel's sign and Phalen's tests are two physical examinations frequently applied to assess CTS following the observation of abnormal sensory responses using provocative maneuvering [3]. Needle electromyography and nerve conduction measurements are two relatively objective modalities used to diagnose CTS [4,5]. Although they show good sensitivity and specificity for CTS diagnosis, the nerve conduction modalities are still not capable of providing appropriate anatomical information for discerning variations of the MN and surrounding tissues in the carpal tunnel.

To date, noninvasive imaging modalities, such as magnetic resonance imaging (MRI) and ultrasound imaging have been applied to acquire anatomical information in the carpal tunnel for assessing CTS [2,6,7]. In general, clinical use of MRI images offers better spatial resolution and higher contrast than those of ultrasound images, which uses transducer frequencies of less than 10 MHz for imaging soft tissues in the MN and surrounding flexor tendons [6]. Yet, the MRI modality, in addition to being less cost-effective, involves a relatively time-consuming imaging procedure. This limits the wide application of MRI in the preliminary or pre-screening evaluation of CTS in clinical diagnosis. On the other hand, ultrasound imaging is broadly accepted due to its non-invasiveness, real-time imaging, non-ionization, and cost-effectiveness. With these advantages, several measurements were performed to utilize ultrasound images to detect the pathological changes of the MN in the wrist tissues, in which diverse parameters including echogenicity, cross-sectional area and carpal tunnel, flattening ratio, and thickness and bowing of the flexor retinaculum were observed [8–12]. Using the cross-sectional areas of the MN ranging from 9 to 12 mm^2^ as a criterion, the sensitivity and specificity of CTS diagnosis were able to reach up to 80% [12–16]. Nevertheless, it remains difficult to establish a certain cutoff value for accurately assessing CTS only using the information of the cross-sectional area of the MN.

MN gliding in patients was further found to be hindered by fibrosis of the subsynovial connective tissue (SSCT) [17–19]. Therefore, in addition to the morphological information, the kinetic trajectory of the MN in the carpal tunnel could also be adopted for assessing CTS. A preliminary study has been performed to further investigate the correlation between the motion of fingers, displacements of the MN, and flexor tendons [20]. It revealed that the displacement between the flexor digitorum superficialis tendon and the MN in an asymptomatic wrist with long-finger flexion tended to significantly increase in the palmar-dorsal direction and decrease in the ulnar-radial direction compared to those resulting from fist flexion [19]. To comply with the index finger moving from full extension to flexion, the corresponding tendon tended to move slightly toward the palmar side in which the MN slides to the radius in the asymptomatic wrist [20]. The MN and tendon moved toward the ulnar and palmar sides, respectively, in response to thumb flexion [20]. Moreover, the MN in the asymptomatic wrist was discerned to slip away from the flexor digitorum superficialis of the index finger and flexor pollicis longus tendons to avoid being compressed in between the tendons and flexor retinaculum [20]. In addition, as the metacarpophalangeal joint was extended from 90° to 0°, the MN in the forearm was moved longitudinally some 2.62 mm for both the asymptomatic and CTS subjects [21]. Although these just mentioned studies generally demonstrated changes of the displacement of the MN and flexor tendons that correspond to the movements of fingers to their full extension and flexion, thorough information about the kinetic trajectories of the MN and flexor tendons in response to the flexion and extension of the fingers it is still lacking.

Furthermore, the abovementioned studies generally utilized ultrasound frequencies between 10 and 17 MHz that only allow limited image resolution and sensitivity to discern tissues in the carpal tunnel for the CTS diagnosis. Certainly, it is straightforward to improve the sensitivity of CTS diagnosis by increasing the frequency and resolution of the employed ultrasound [22]. To date, ultrasounds with frequencies higher than 20 MHz have been verified to be appropriate for measuring various fine tissues, such as the skin, eye, vasculature, blood, and small animals [23–28]. All of those previous works were consolidated in the current study to further develop a 30 MHz high-frequency ultrasound system for measuring variations of tissue structure and the displacement characteristics of the MN in the wrist corresponding to movements of fingers at different flexion angles. A two-dimensional kinetic trajectory of the MN in the carpal tunnel of the wrist was then detected by a cross correlation-based motion tracking technique.

## 2. Experimental Section

The experiment was carried out on a total of fourteen asymptomatic volunteers, including seven males and seven females, with a mean ± standard deviation age of 24.6 ± 2.73 years. Participants with a history of CTS, wrist surgery, tenosynovitis, rheumatoid arthritis, osteoarthritis, degenerative joint disease, acromegaly, diabetes, hypothyroidism, amyloidosis, gout, or traumatic injuries to the hand or wrist were screened and excluded for the present study [19]. All of participants gave consent for the testing after detailed descriptions about the experimental objective and procedures were given. This study protocol was approved by the institutional review board of National Cheng Kung University Hospital in Tainan, Taiwan.

### 2.1. Experimental Arrangements

**Figure 1.**
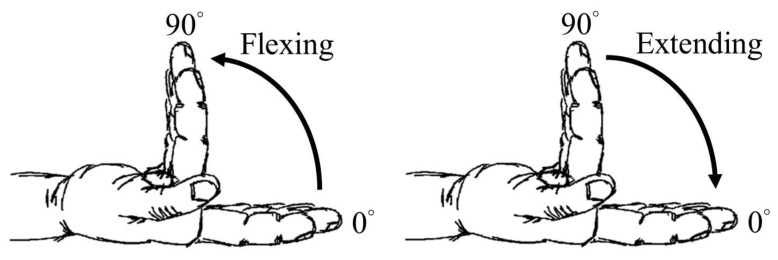
Schematic illustrations of the movements of four fingers during flexion and extension.

The subject was asked to outstretch the right arm with the palm up and the elbow flexed to 90° with the forearm during each experiment. Subsequently, the forearm was fixed to the bottom of a custom-made plexiglass tank filled with distilled water for ultrasound imaging. The four fingers (index, long, ring, and little) were maintained in the extension direction using a plate positioned on the dorsum aspect of the interphalangeal joints and a Micropore^TM^ tape placed across the proximal interphalangeal joints. The measured position was approximately at a 7.5 mm away from the level of the wrist crease. The ultrasonic radio-frequency (RF) signals and images of the wrist in the transverse-plane were collected, in which data corresponding to the flexion and extension movements of four fingers from 0° to 90° and 90° to 0°, respectively, with an increment angle of 5°, were acquired, as shown in Figure 1. The flexion angle of four fingers fixed on a plate was better controlled by a micro-stepping rotatory motor. A total of five repeat experiments for each subject covering movements between flexion and extension were performed.

**Figure 2.**
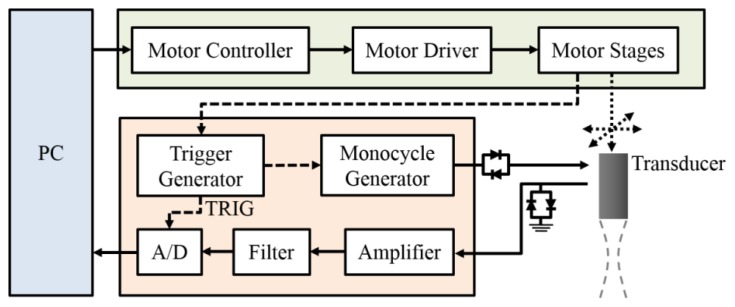
Schematic diagram of the high-frequency ultrasound system.

A schematic diagram detailing the arrangement of high-frequency ultrasound system is shown in Figure 2, in which a 30 MHz single-element ultrasound transducer (NIH Ultrasonic Transducer Resource Center, USC, Los Angeles, CA, USA), with the acoustic characteristics summarized in Table 1, was employed for the generation and reception of ultrasound waves.

A high-voltage monocycle generator (AVB2-TB-C, AVTECH Electrosystems Ltd., Ogdensburg, NY, USA) was used to generate a 200 peak-to-peak voltage for the exciting transducer, and which was connected to an electronic expander (Matec Instruments Company, Northborough, MA, USA) to eliminate electrical noise. The received ultrasonic RF signals were connected to an electronic limiter (Matec Instruments Company) to protect the device from damage and then they were amplified by a low-noise amplifier (Model LN1000A, Amplifier Research, Souderton, PA, USA). Subsequently, signals were filtered by a bandpass filter (Model BIF-30, Mini-Circuits, New York, NY, USA) and then digitized by an 8-bit analog-to-digital converter (PXI 5152, National Instruments, Austin, TX, USA) at 200 MHz sampling frequency. The transducer was mounted on the piezoceramic motor (HR8, Nanomotion Ltd., Yokneam, Israel) for sweep scanning the image of the wrist at different locations in the transverse plane. In addition, the transducer was also allowed to flexibly move to two other perpendicular directions along the two axes of micro-stepping motors (CM1-C-17L30A, Cool Muscle, Osaka, Japan) equipped with actuators (KR2602A, THK, Tokyo, Japan). All of these motion stages were controlled by a motor controller (DMC-1842, Galil Motion Control Inc., Rocklin, CA, USA). The program for data acquisition and motor control was developed using LabVIEW software (National Instruments, Austin, TX, USA). The mean signal-to-noise ratio of acquired ultrasonic signals was 36 dB. The acquired digital signals were processed with a sequence of filtering, Hilbert transform, logarithmic compression, and gray scale mapping to form B-mode images with 42 dB dynamic range [29]. The size of a B-mode image is 15 mm × 7.5 mm, which is composed of 750 A-lines with 20 μm intervals.

### 2.2. Analysis of Kinetic Trajectory

**Figure 3.**
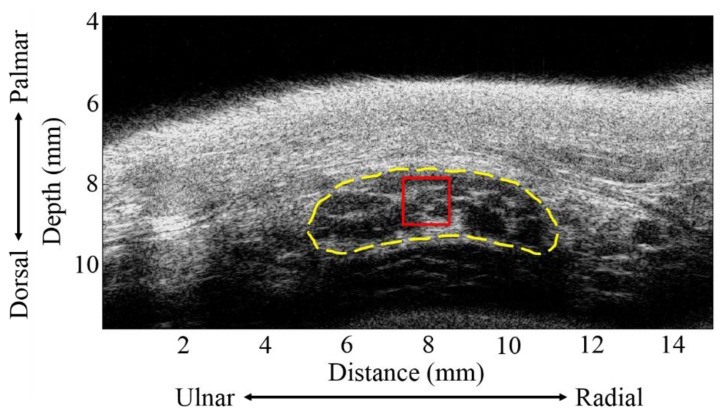
B-mode images of the MN. The yellow contour drawn manually corresponds to the MN. The red rectangle indicates region of interest for motion tracking.

The kinetic trajectory of the MN was analyzed offline from all of the acquired images of each subject by applying a normalized cross-correlation to consecutive B-mode images corresponding to adjacent angles of finger movement. The analysis begins with manually selecting a region of interest (ROI) image that covers the MN from the first of sequence images associated with 0° flexuous angle of fingers as the kernel pattern, as shown in Figure 3. The size of kernel pattern was approximately 1.2 mm × 1.2 mm. Subsequently, the search pattern on the following frame of image was acquired at the adjacent angle aspect of reference image, and which was selected automatically corresponding to the position of kernel pattern. The cross-correlation coefficient (ρ) between the kernel pattern and search pattern was calculated by:

(1) $$\rho_{n,m}(k,l)=\frac{\overset{K/2}{\underset{i=-K/2}{\text{∑}}}\overset{L/2}{\underset{j=-L/2}{\text{∑}}}\left\{[F_{1}(n+i,m+j)-\bar{F_{1}}][F_{2}(n+i+k,m+j+l)-\bar{F_{2}}]\right\}}{{\left[\overset{K/2}{\underset{i=-K/2}{\text{∑}}}\overset{L/2}{\underset{j=-L/2}{\text{∑}}}{\left[F_{1}(n+i,m+j)-\bar{F_{1}}\right]}^{2}\right]}^{1/2}{\left[\overset{K/2}{\underset{i=-K/2}{\text{∑}}}\overset{L/2}{\underset{j=-L/2}{\text{∑}}}{\left[F_{2}(n+i+k,m+j+l)-\bar{F_{2}}\right]}^{2}\right]}^{1/2}}$$

where *F*_1_ and *F*_2_ represent respectively the reference and following frame of images, *n* and *m* denote the corresponding two-dimensional positions of a kernel pattern, *k* and *l* are the coordinates of trial matching region in *F*_2_, *K* × *L* correspond to the size of a kernel patter, and *F̅*_1_ and *F̅*_2_ are the mean values of the corresponding kernel pattern and search pattern, respectively. The size of each trial matching region was 8 mm × 5 mm based on the position of the kernel pattern. The position of the MN on the following frame of image was found as a maximum correlation coefficient between the kernel pattern and search pattern was achieved. Consequently, the search pattern was recorded as a new kernel pattern for subsequently tracking the position of the MN on the following images.

The displacements of the MN, corresponding to the flexuous angle positioned at 0°, in the ulnar-radial (*UR*) and palmar-dorsal (*PD*) directions were analyzed. The positive displacement corresponds to the MN movement toward either the radial or palmar direction, whereas the negative displacement is the MN movement toward either ulnar or dorsal direction. The incremental displacements of the MN in the *UR* and *PD* directions relative to those previously adjacent flexuous angles were defined as *VUR* and *VPD*, respectively, and which were calculated by:

(2) $$VUR_{i}=UR_{i}-UR_{i-1}$$

and:

(3) $$VPD_{i}=PD_{i}-PD_{i-1}$$

where the suffix *i* denotes the number of fingers movement with each incremental value of 1 meaning that the increment of the flexuous angle is 5°. The resultant displacement (*R*, displacement in total direction) calculated with respect to the MN positioned at 0° of flexuous angle was further analyzed accordingly to the polar coordinate system, given as:

(4) $$R_{i}=\sqrt{{\left(PD_{i}\right)}^{2}+{\left(UR_{i}\right)}^{2}}$$

The direction angle (*φ*) of movement was given by:

(5) $$\phi_{i}=\tan^{-1}\left(\frac{PD_{i}}{UR_{i}}\right)$$

The incremental displacements (*VR*) in a total direction between the adjacent flexion angles was calculated by:

(6) $$VR_{i}=\sqrt{{\left(VUR_{i}\right)}^{2}+{\left(VPD_{i}\right)}^{2}}$$

The algorithm for ultrasonic imaging, motion tracking, and displacement analysis were implemented by MATLAB software (The MathWorks, Natick, MA, USA). The t-test was applied to study the significant difference of displacement of the MN between male and female. The *p*-value smaller than 0.05 was considered to be significant.

## 3. Results

**Figure 4.**
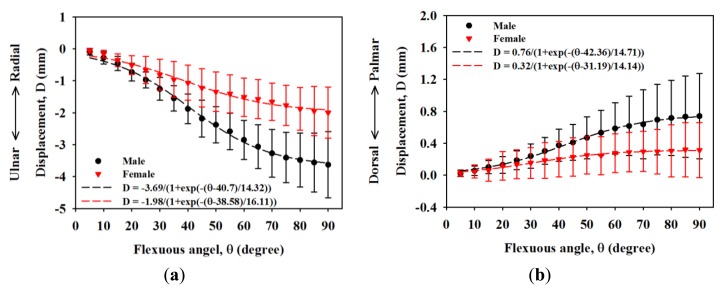
Displacements of the MN in the (**a**) ulnar-radial direction and (**b**) dorsal-palmar direction for males and females while flexing fingers from 0° to 90°.

Figure 3 shows a typical high-frequency ultrasound image detailing the internal structure of the MN which is as shown within the manually drawn yellow contour that is composed of the hypoechoic region with discontinuous bands. The displacements of the MN in the ulnar-radial and palmar-dorsal directions corresponding to the movement of fingers are shown in Figure 4. The MN tends to move toward the ulnar and palmar directions with respect to the flexion of fingers from 0° to 90°. The displacement of the MN in the ulnar-radial direction is generally larger than that in the palmar-dorsal direction. The MN displacements in the ulnar-radial direction as a function of flexion angle were best fitted with a sigmoidal equation, in which R-squares for data of male and female subjects were 0.82 and 0.58, respectively. The displacements of the MN in the ulnar-radial direction for males corresponding to the flexion angles larger than 35° were significantly larger than those of females (*p* < 0.05). As the flexion angle moved towards 90°, the displacements in the ulnar-radial direction for males and females were measured to be −3.63 ± 1.03 mm and −1.99 ± 0.80 mm, respectively, and those of the displacements in the palmar-dorsal direction were 0.74 ± 0.53 mm and 0.31 ± 0.34 mm, respectively. The MN displacements in the palmar-dorsal direction between male and female subjects was not significantly different as that of in the ulnar-radial direction. Moreover, the trajectory for the displacement of the MN in the palmar-dorsal direction as a function of flexion angle does not follow a sigmoidal tendency with the R-squares of fitted curves for male and female subjects of 0.42 and 0.16, respectively.

**Figure 5.**
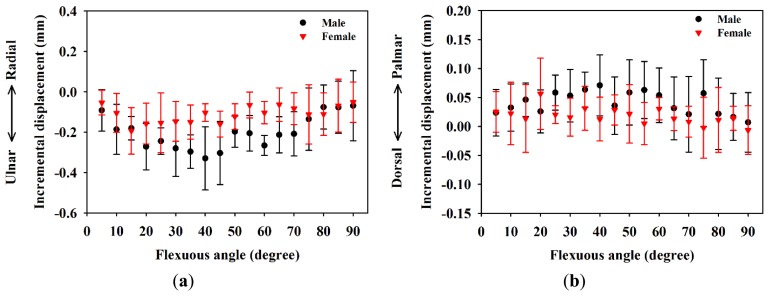
Incremental displacements of the MN in the (**a**) ulnar-radial direction and (**b**) dorsal-palmar direction in males and females while flexing fingers from 0° to 90°.

Following the movements of fingers flexion from 0° to 90°, male subjects show incremental displacements of the MN in the ulnar-radial direction between −0.6 mm and 0.2 mm, and those of female subjects are between −0.4 mm and 0.1 mm, as shown in Figure 5a. The average incremental displacement of the MN for male subjects (−0.20 ± 0.06 mm) in the ulnar-radial direction is significantly larger than that of female subjects (−0.11 ± 0.04 mm), where the *p*-value is 0.006. It also may be observed that the incremental displacements for male subjects are larger than those of females associated with the flexion angles between 30° and 70°. In response to the fingers flexing from 0° to 90°, the incremental displacements of the MN in the palmar-dorsal direction were measured to be between −0.12 mm and 0.17 mm, and −0.08 mm and 0.16 mm for male and female subjects, respectively, as shown in Figure 5b. The average incremental displacement of the MN in the palmar-dorsal direction did not show a significant difference between male (−0.041 ± 0.030 mm) and female (−0.018 ± 0.020 mm) subjects, with a *p*-value of 0.107.

**Figure 6.**
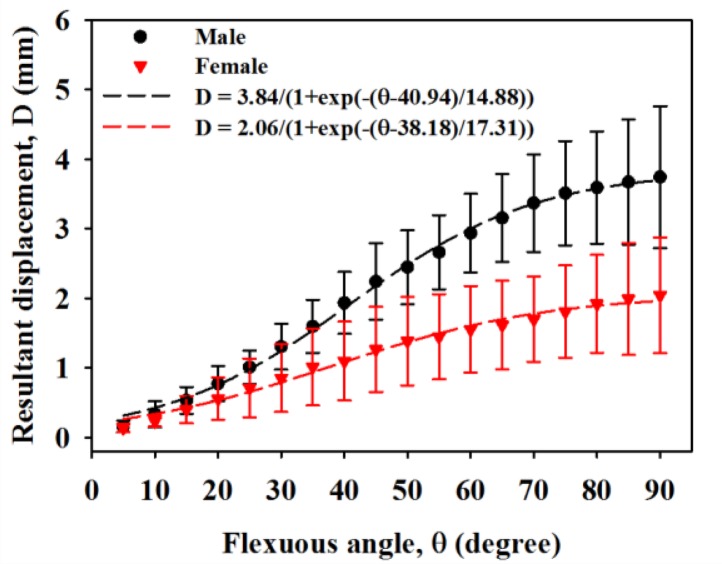
Resultant displacements of the MN in males and females while flexing fingers from 0° to 90°.

**Figure 7.**
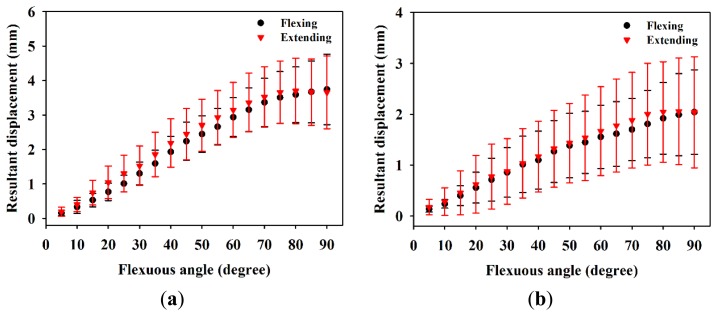
Resultant displacements of the MN while flexing and extending fingers in (**a**) males and (**b**) females.

**Figure 8.**
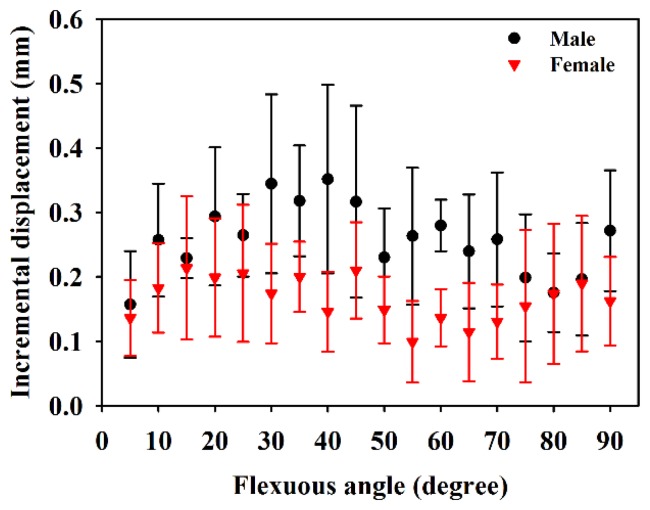
Incremental displacements of the MN in total direction for males and females while flexing fingers from 0° to 90°.

Figure 6 is the resultant displacement of the MN as a function of flexion angle, which shows that the displacement of male subjects in general increases faster than that of females. The kinetic trajectory of resultant displacement during finger flexing also generally follows the sigmoidal model with R-squares of 0.84 and 0.56 for male and female subjects, respectively. When the flexion angles is larger than 35°, the resulting displacements of the MN for male subjects tended to be much larger than those of females (*p* < 0.05). As the four fingers moved to 90°, the corresponding resulting displacements for male and female subjects were estimated to be 3.74 ± 1.02 mm and 2.04 ± 0.83 mm, respectively. Figure 7 shows the resulting displacements of the MN for male and female subjects as a function of flexion angle corresponding to flexing and extending finger movements, in which the resulting displacements are quite consistent between both movements. The incremental displacements of the MN in the total direction were found to range between 0.05 mm and 0.6 mm and between 0.02 mm and 0.4 mm for males and females, respectively, corresponding to the flexing of fingers from 0° to 90°, as shown in Figure 8. The average increment of the resulting displacement of the MN for males (0.26 ± 0.05 mm) was significantly larger than that for females (0.17 ± 0.04 mm) with a *p*-value of 0.005. The incremental displacements for males were larger than that for females for flexion angles between 30° and 70°.

**Figure 9.**
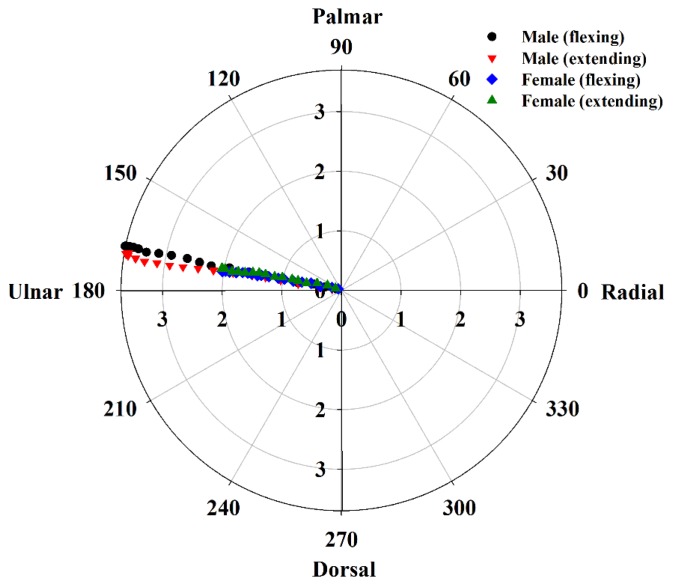
Averaged kinetic trajectories of the MN during finger flexion and extension in males and females.

Furthermore, the average kinetic trajectories of the MN associated with flexion and extension movements of fingers for males and females are shown in Figure 9, in which the MN tends to move toward a constant direction following finger flexion or extension movements. The angles of movement direction of the MN for males were 167.9° ± 1.0° and 167.2° ± 8.5° during flexion and extension of fingers, respectively, and those of females were 168.7° ± 6.0° and 165.7° ± 6.7°, respectively In addition, both the MNs for males and females tended to show similar kinetic trajectories in response to finger movements between flexion and extension.

## 4. Discussion

In accordance with the movement of fingers, the flexor tendon moves actively to transfer the force from the muscle to the phalanx bone, while that of the MN is passively moved following the indirect traction by the flexor tendons, which is mediated by the SSCT to avoid the compression from tendons. The SSCT consists of multiple layers of collagen fibers with a certain amount of the blood and lymphatic vessels [17]. The SSCT functions to reduce friction to protect the blood supply for the tendons and synovium associated with tendon motion [30]. Recently, several studies have demonstrated that patients with CTS tend to have fibrosis and thickening of the SSCT as evidenced by histological analysis [17,31]. The fibrosis and thickening of the SSCT may restrict the MN from sliding out of the way during finger motion [19,20,32]. Therefore, it provides the possibility for assessing CTS by measuring the kinetic information of the MN. In the present study, the relationship between the flexion angles of fingers and the corresponding displacements of the MN was explored from measurements using taken on 14 asymptomatic human volunteers. The kinetic trajectory of the MN corresponding to simultaneous movement of four fingers, which is able to compress the MN by tendons in the carpal tunnel, was measured using high-frequency ultrasound [19,21]. To improve the accuracy and repeatability of the experiment, a rotational plate controlled by a micro-stepping motor was developed to flexibly adjust finger movements from 0° to 90° motion, and two-dimensional motion tracking was applied to analyze the displacement of the MN from the acquired ultrasound images. Experimental results showed that the MN moved toward the ulnar and palmar directions corresponding to the movements of fingers flexing from 0° to 90°, and which were consistent with those obtained by Yoshii *et al.* [19]. The resultant displacement of the MN was 2.89 ± 1.26 mm (an average of the displacement of the MN from male and female subjects), which is a reasonable result comparable to those reported in previous study [19] where motion of fist fingers was performed. In addition, as the flexion angles became larger than 35°, both the displacements of the MN in the ulnar-radial direction and total direction measured from male subjects were significantly larger than those of female subjects. When the flexion angle was adjusted to be between 30° and 70°, both the incremental displacements in the ulnar-radial direction and total direction for males were also larger than those of females. The dimension and movement of the flexor tendon as well as the elasticity of SSCT may influence the displacement of the MN according to the movement of fingers due to the fact that the displacement of the MN represents an indirect traction by the flexor tendons. Carroll *et al.* [33] indicated that the cross-section area of the patellar tendon in men (131.9 mm^2^) was larger than that of women (103.1 mm^2^). Nevertheless, the dependency of cross-section area of the digital flexor tendon on gender has not yet been reported. It therefore is worthy to studying the influence of the dimension of the digital flexor tendon on the displacement of the MN with respect to finger movement. Furthermore, both the displacements of the MN in the ulnar-radial direction and total direction are sigmoidal tendencies with respect to the angle of flexion movement. The tendencies allow the implementation of basic sigmoidal models to further simulate the kinetic trajectory of the MN with respect to finger movements. Other factors, such as the size of both the MN and tendon, tendon movement, elasticity of SSCT, could affect the gliding of the MN and those are worthy of further investigation to improve the trajectory models. Nevertheless, the current study validates that the implementation of both high-frequency ultrasound and motion tracking techniques are capable of sensitively detecting the kinetic trajectory of the MN corresponding to flexion and extension movements of the fingers. Results obtained from asymptomatic subjects may be used as a reference for further comparison with those of from patients with CTS.

In the present study, measurements of the kinetic trajectory of the MN corresponding to the flexion and extension movements of fingers were carried out mainly in the traverse-plane. In addition to gliding in the traverse-plane, the MN would also glide to the longitudinal direction during finger movement [21]. This may lead the kernel region of the MN, which corresponds to the acquired B-mode images of flexing fingers at a 0° angle, to be out of range the view of imaging at the next flexion angle. Nevertheless, the movement analysis of the MN could still be performed using two-dimensional motion tracking because there is little variation for image patterns of the MN between two consecutive B-mode images of an adjacent angle movement of fingers. To further improve the assessment accuracy, three-dimensional measurement and motion tracking may be essential to analyze the gliding of the MN in the transverse and longitudinal planes. Furthermore, the ultrasound images were acquired at the proximal edge of the carpal tunnel rather than that of the middle or distal part. Although distal images may be useful to observe the MN compression, imaging the carpal tunnel at the distal part requires exerting a certain pressure on the relatively thicker subcutaneous tissues of the palm by the transducer [19]. The exerted pressure may also affect the motion of the subjacent MN and flexor tendons. In addition, it is still difficult for the employed 30 MHz high-frequency ultrasound to penetrate into relatively thicker subcutaneous tissues and transverse carpal ligaments where several other researchers have shown that the measurement of the MN in the proximal part of carpal tunnel was useful to evaluate the CTS [11,15,19,20]. In comparison with other studies in the literature, the current study tended to assess the kinetic trajectory of the MN with a limitation sample of fourteen asymptomatic volunteers. With sufficient resolution and sensitivity, the customized 30 MHz ultrasound imaging system and computer program could feasibly be applied to clinical trials on patients with CTS. Accordingly, the setup and specification of the ultrasound system as well as the design of the supportive holder for patients' hands could be improved before the whole system would be routinely used in clinical practice.

## 5. Conclusions

A high-frequency ultrasound imaging system with a frequency of 30 MHz was developed and applied to measure the movement of the MN associated with flexion and extension movements of fingers for males and females. The kinetic trajectory of the MN was analyzed using motion tracking based on a normalized cross-correlation. The results indicated the the MN was moved toward the ulnar and palmar directions with respect to the flexion of fingers from 0° to 90°. When the flexion angle was 90°, the resultant displacements for males and females were 3.74 ± 1.02 mm and 2.04 ± 0.83 mm, respectively. The displacements of the MN in the ulnar-radial direction and total direction for males corresponding to flexion angles larger than 35° were distinctly larger than those of females. Both the displacements of the MN in the ulnar-radial direction and total direction may be best fitted to a sigmoidal model with respect to the angle of flexion movement. The angles of movement direction of the MN for males and females were 167.9° ± 1.0° and 168.7° ± 6.0° during fingers flexions, respectively. This study has verified that high-frequency ultrasound imaging and motion tracking are feasible to be utilized for analyzing the displacement of MN during finger motion. The kinetic trajectory of the MN obtained from asymptomatic subjects may be used as a reference for further comparison with that of patients with CTS.

## Figures and Tables

**Table 1.** Characteristics of the applied transducer.

Central frequency	32 MHz
−6 dB band width	18.6 MHz
*f*-number	1.5
Depth of focus	9 mm
Aperture size	6 mm
Axial resolution	32 μm
Lateral resolution	68 μm
